# Impact of HbA1c control and type 2 diabetes mellitus exposure on the oral microbiome profile in the elderly population

**DOI:** 10.1080/20002297.2024.2345942

**Published:** 2024-05-15

**Authors:** Xin Zeng, Shuqi Huang, Xin Ye, Siping Song, Jing He, Liwei Hu, Sicheng Deng, Fan Liu

**Affiliations:** a West China School of Nursing, Sichuan University, Chengdu, China; b Nursing Department, State Key Laboratory of Oral Diseases & National Clinical Research Center for Oral Diseases, West China Hospital of Stomatology, Sichuan University, Chengdu, China; c Post anesthesia Care Unit, West China Hospital of Stomatology, Sichuan University, Chengdu, China; d Department of Oral Mucosal Diseases, West China Hospital of Stomatology, Sichuan University, Chengdu, China; e Department of Oral Surgery, West China Hospital of Stomatology, Sichuan University, Chengdu, China; f Innovation Center of Nursing Research, Nursing Key Laboratory of Sichuan Province, Chengdu, China

**Keywords:** Elderly individuals, type 2 diabetes mellitus, oral microbiome, glycated haemoglobin, diabetes duration

## Abstract

**Objective:**

To investigate the associations of the oral microbiome status with diabetes characteristics in elderly patients with type 2 diabetes mellitus.

**Methods:**

A questionnaire was used to assess age, sex, smoking status, drinking status, flossing frequency, T2DM duration and complications, and a blood test was used to determine the glycated haemoglobin (HbA1c) level. Sequencing of the V3-V4 region of the 16S rRNA gene from saliva samples was used to analyze the oral microbiome.

**Results:**

Differential analysis revealed that *Streptococcus* and *Weissella* were significantly enriched in the late-stage group, and *Capnocytophaga* was significantly enriched in the early-stage group. Correlation analysis revealed that diabetes duration was positively correlated with the abundance of *Streptococcus* (*r*= 0.369, *p*= 0.007) and negatively correlated with the abundance of *Cardiobacterium* (*r*= -0.337, *p*= 0.014), and the level of HbA1c was not significantly correlated with the oral microbiome. Network analysis suggested that the poor control group had a more complex microbial network than the control group, a pattern that was similar for diabetes duration. In addition, Streptococcus has a low correlation with other microorganisms.

**Conclusion:**

In elderly individuals, *Streptococcus* emerges as a potential biomarker linked to diabetes, exhibiting elevated abundance in diabetic patients influenced by disease exposure and limited bacterial interactions.

## Introduction

Type 2 diabetes mellitus (T2DM) is a metabolic disorder characterized by chronic hyperglycemia caused by insulin resistance [1]. It is the most common form of diabetes mellitus in clinical practice, accounting for approximately 85–90% of all cases [2]. Globally, it has a high prevalence of disability, death and economic burden worldwide [3,4]. Aging is associated with progressive impairment of insulin secretion and increased insulin resistance [5]. Therefore, the importance of preventing and managing T2DM in elderly individuals is receiving increasing attention as the global population ages.

Various studies have shown that an unbalanced microbiome is associated with the development of dental caries, periodontal disease, T2DM and circulatory disorders [6]. Apart from the gut microbiome, the oral microbiome is the most diverse microbiome in the human body and is colonized by more than 700 species of bacteria [7]. The oral microbiome has been found to play an important role in triggering and exacerbating T2DM [8,9]. First, the transfer of the oral microbiome to the liver can restrain glycogen synthesis via the MAP kinase signalling pathway and the Akt/GSK-3β signalling pathway, ultimately leading to insulin resistance [10,11]. Second, the oral microbiome can secrete lipopolysaccharide into the bloodstream, increasing the expression of inflammatory factors (IL-1, TNF-α, etc.) through a series of pathways, leading to chronic low-grade inflammation throughout the body, and inflammatory factors can directly or indirectly act on pancreatic islet cells, affecting insulin secretion and causing insulin resistance [12,13]. Third, the oral microbiome is strongly associated with obesity and may play an important role in the etiology of T2DM [14,15]. In addition, T2DM can also change the oral microbiome. Patients with poorer glycemic control have elevated levels of glucose in their saliva, which alters the availability and concentration of nutrients needed for bacterial growth and alters their diversity [12]. Inflammatory factors are upregulated in patients with T2DM, where increased IL-17 production alters the pathogenicity of the oral microbiome and promotes oral diseases such as periodontitis and caries [16,17]. With advances in sequencing technology, sequencing is faster and more accurate and will provide a wider range of microbial information for the development of microbial markers, providing new ideas for the prediction, diagnosis and even treatment of T2DM [18,19].

Although the specific oral microbiome for each disease is controversial, the oral microbiome differed between disease groups and healthy controls, as manifested by a decrease in health-associated bacterial taxa and an increase in bacterial taxa associated with disease [9]. Changes in the oral microbiome of diabetic patients and healthy controls have been studied more extensively; however, a wide age range of subjects was included. One study [20] analysed the salivary microbiome of elderly and younger adults and revealed that alpha diversity was significantly greater in elderly adults than in younger adults, suggesting that age influences the composition of the oral microbiome. However, there are few studies on the elderly population, with only one Spanish study [21] and two Japanese [5,20] studies, and there is a lack of exploration of the oral microbiome in elderly Chinese patients with T2DM.

A study on the effect of glucose control on the oral microbiome, with indicators including fasting blood glucose (FBG), salivary glucose (SG) and glycated haemoglobin (HbA1c), revealed that hyperglycaemia was correlated with the proportions of *Treponema*, *Desulfobulbus*, *Phocaiecola* and *Saccharimonadaceae* [22]. In addition to FBG, SG and HbA1c, diabetes duration and complications are also strongly associated with T2DM progression [23]. Moreover, oral hygiene, dietary habits, smoking status, drinking status, age, medication, health status, genetics, environmental exposures, and socioeconomic status all influence the composition of the microbiome [24–27]. However, the above oral microbiological influences have rarely been explored in elderly patients with T2DM. Therefore, this study will further investigate the effects of age, oral hygiene, lifestyle, and diabetes-related characteristics (glycaemic control, T2DM duration and complications) on the oral microbiome of elderly patients with T2DM and provide a basis for further exploration of oral microbiological markers in elderly patients with T2DM.

## Methods

### Study design and participants

This cross-sectional study was conducted between August 2022 and January 2023 at a dental hospital, which is the National Centre for Stomatology and the National Clinical Medical Research Centre for Oral Diseases. The study was performed according to the Declaration of Helsinki and was approved by the Ethics Committee of West China Hospital of Stomatology Sichuan University with approval number WCHSIRB-D-2022-291. All participants signed an informed consent form. Before the study, all researchers undertook homogenized training and assessments to ensure the quality of the study.

The inclusion criteria were as follows: 1) aged ≥60 years; 2) met the diagnostic criteria for T2DM (self-reported); 3) had ≥ 20 natural teeth; and 4) voluntary participation. The exclusion criteria were as follows: 1) cognitive dysfunction and psychiatric disorders; 2) physical dysfunction; 3) systemic use of antibiotics and immunosuppressants in the last 6 months; 4) high-dose use of probiotics in the last 6 months; 5) use of topical antibiotics in the last 7 days; 6) periodontitis stage III and IV [28]; 7) oral tumours; 8) frequent episodes of hypoglycaemia or cardiovascular disease in the last 3 months; and 9) restriction of mouth opening. Each study participant completed a questionnaire on age, sex, smoking status, drinking status, frequency of flossing, T2DM duration and T2DM complications, and HbA1c values were obtained via venous blood tests.

### Saliva collection

(i) Study participants were told not to eat, drink, smoke or chew gum at least 2 hours before sampling; (ii) upper and lower jaws were scraped as many times as possible with the tongue before saliva was collected and the tongue was scraped slightly with the teeth; (iii) when the participants assumed a seated position, they leaned forward and lowered the head, relaxed and massaged the cheeks, and the saliva would flow into the funnel until the amount of saliva (without air bubbles) was ≥2 ml; (iv) after sampling, the collected salivary tubes were immediately numbered and placed in an ice box and transferred to a −80°C refrigerator for storage within 2 hours.

### DNA extraction and 16S rRNA gene amplicon sequencing

DNA extraction was performed according to the directions of the E.Z.N.A.® soil DNA kit (Omega Biotek, Norcross, GA, U.S.). The quality of the DNA was checked by agarose gel electrophoresis with 1% agarose, and the concentration and purity of the DNA were determined using a NanoDrop2000 (Thermo Scientific, U.S.A.).

PCR amplification of the V3-V4 variable region of the 16S rRNA gene was performed using the upstream primer 338F (5’-ACTCCTACGGGGAGGCAGCAG-3“) and the downstream primer 806 R (5”-GGACTACHVGGGGTWTCTAAT-3’). The PCR system included 4 μL of 5× TransStart FastPfu buffer, 2 μL of 2.5 mM dNTPs, 0.8 μL of upstream primer (5 µM), 0.8 μL of downstream primer (5 µM), 0.4 μL of TransStart FastPfu DNA polymerase, 0.2 μL of BSA, 10 ng of template DNA, and ddH2O to 20 μL. The PCR amplification procedure was as follows: predenaturation at 95°C for 3 min; 30 cycles of denaturation at 95°C for 30 s, annealing at 53°C for 30 s, and extension at 72°C for 45 s; a stable extension at 72°C for 10 min; and storage at 10°C until the reaction was complete. There were 3 PCR replicates per sample, the PCR products from the 3 replicates were mixed, and the products were detected using 2% agarose gel electrophoresis. PCR products were purified using the AxyPrep DNA Gel Extraction Kit (Axygen Biosciences, Union City, CA, USA) and quantified using a Quantus™ Fluorometer (Promega, USA). A MiSeq library was constructed using a NEXTFLEX Rapid DNA-Seq Kit (Bioo Scientific, USA).

### Sequencing analysis

High-throughput sequencing was performed using Illumina MiSeq PE300 platform (Shanghai Meiji Biomedical Technology Co.). The original sequences were subjected to quality control (QC) using fastp [29] (https://github.com/OpenGene/fastp, version 0.20.0) software, and FLASH [30] (http://www.cbcb.umd.edu/software/flash, version 1.2.7) software was used to extend the lengths of short reads by identifying overlaps between paired-end reads. After QC splicing, the sequences were subjected to operational classification unit (OTU) clustering, and chimeras were removed based on 97% similarity using UPARSE [31] software (http://drive5.com/uparse/, version 7.1). Taxonomic annotation of OTU species was performed using the RDP classifier [32](http://rdp.cme.msu.edu/, version 2.11) compared to the Silva 16SrRNA gene database (v138).

### Data analysis

The Chao 1 index, Shannon index, etc., were calculated using mothur [33] software (http://www.mothur.org/wiki/Calculators), and Student’s t test or one-way ANOVA was used for the analysis of between-group differences in alpha diversity. The Bray‒Curtis distance-based algorithm-based PCoA analysis (principal coordinate analysis) was used to test the similarity of microbial community structure between samples, and the ANOSIM nonparametric test was used to analyse whether the differences in microbial community structure between sample groups were significant. Linear discriminant analysis effect size (LEfSe) [34] (http://huttenhower.sph.harvard.edu/LEfSe) (LDA >3, *p* < 0.05) was used to identify bacterial taxa with significant differences in abundance from phylum to genus level among different groups. Spearman’s correlation was used to analyse the correlation of environmental factors with the microbiome and the relationships between microorganisms.

## Results

### Characteristics of the participants

Fifty-two elderly T2DM patients were recruited through a poster combined with a family doctor’s presentation. Clinical characteristics of all participants are shown in Table 1 and Supplementary Table T1.

**Table 1.** Demographic and clinical characteristics of all participants.

	All participants (*n*=52)	Number of smokers
**Age-No.(%)**		
Old(60~74y)	39(75)	
Older(≥75y)	13(25)	
**Gender-No.(%)**		
Male	28(53.8)	
Female	24(46.2)	
**Smoking status-No.(%)**		
Nonsmoker*	47(90.4)	
Smoker	5(9.6)	
**Drinking status-No.(%)**		
Heavy drinking	21(40.4)	
Moderate drinking	26(50.0)	
No drinking	5(9.6)	
**Flossing frequency-No.(%)**		
Per day	8(15.4)	
Sometimes	15(28.8)	
Never	29(55.8)	
**HbA1c-No.(%)**		
Control(<7%)	35(67.3)	5(100)
Poor control(≥7%)	17(32.7)	0(0)
**Year-No.(%)**		
Early stage(≤10y)	29(55.8%)	2(40)
Late stage(>10y)	23(44.2)	3(60)
**Complications-No.(%)**		
Yes	14(26.9)	
No	38(73.1)	

*The nonsmokers included never smokers and former smokers.

### Sequencing summary

A total of 2,777,825 raw reads were obtained, with an average of 53,419.7 reads per sample. After quality filtering and mitochondrial and chloroplast removal, 2,776,184 reads remained for further analysis, averaging 53,388 reads per sample. After extraction according to the minimum number of sample sequences 35,314 reads were obtained per sample. Species annotation statistics revealed 15 phyla, 197 genera, 417 species and 569 OTUs. The original relative percentage abundance statistics for the number of sample sequences at each taxonomic level are shown in Supplementary Tables T2 , T3, T4, and T5.

### Composition of the oral microbiome

**Figure 1.**
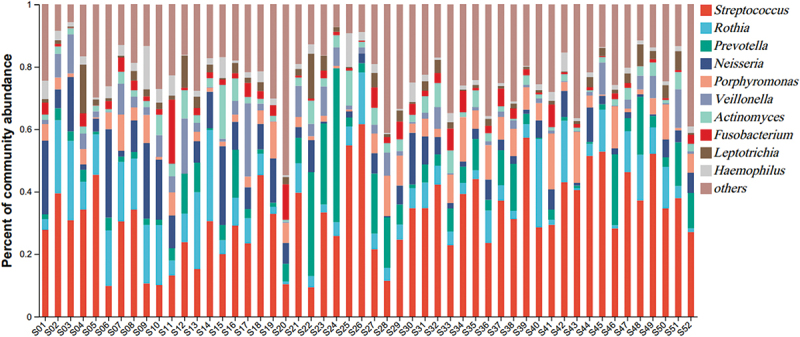
Community bar plot for each sample at the genus level.

At the phylum level, *Firmicutes, Bacteroidota, Actinobacteria, Proteobacteria*, and *Fusobacteria* were the five most abundant phyla (95.74%). At the genus level, *Streptococcus, Rothia, Prevotella, Neisseria*, and *Porphyromonas* were the top five genera at 62.21%, and *Streptococcus* was the top genus at 32.23% (Figure 1). At the species level, s_*unclassified_g__Streptococcus, Rothia_mucilaginosa, Neisseria_subflava, uncultured_organism_g__Veillonella* and *Porphyromonas_gingivalis* were the five most abundant species at 49.52%, and s_*unclassified_g__Streptococcus* was the most abundant species at 31.31%.

### Age, flossing frequency and lifestyle

Smoking and drinking status were not included in the analyses because of the unbalanced sample size gap between the groups, which may have affected the results. We assessed the influence of age and flossing frequency on microbiome diversity in elderly T2DM patients. The results showed that the old group had a greater Shannon index than did the older group, and the difference was statistically significant (Supplementary Table T6). However, no other significant differences in diversity were detected for age or flossing frequency (Supplementary Table T6). The dilution curves showed that the curves tended to parallel, suggesting that the sequencing depth was adequate. In addition, PCoA plots did not show a significant clustering pattern, and ANOSIM analyses showed *p* values > 0.5 in all cases.

### HbA1c, duration of T2DM and complications

#### Microbiological diversity

**Figure 2.**
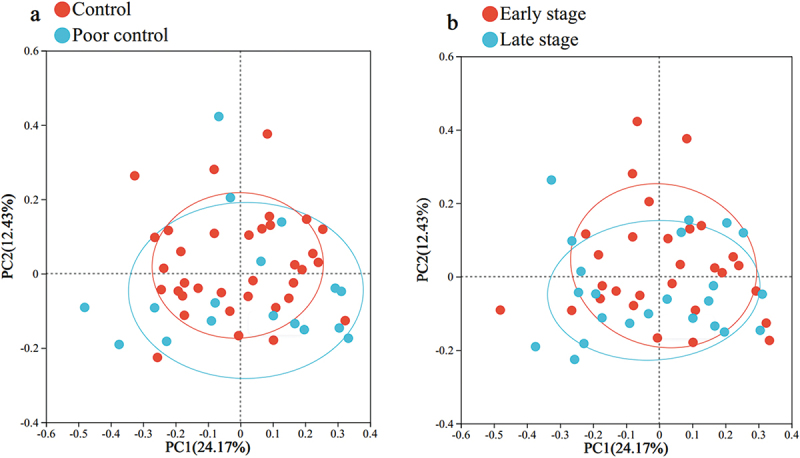
PCoA plot of HbA1c (a) and T2DM duration (b) at the OTU level. The colors represent the different groups, and the dots represent the different samples. The PCoA plot shows the clustering of the two groups.

The results showed no significant difference in the oral microbiome diversity index in elderly T2DM patients in terms of HbA1c, T2DM duration or T2DM complications (Supplementary Table T6). The PCoA plots also did not show significant clustering (Figure 2a, b).

#### Differential analysis

**Figure 3.**
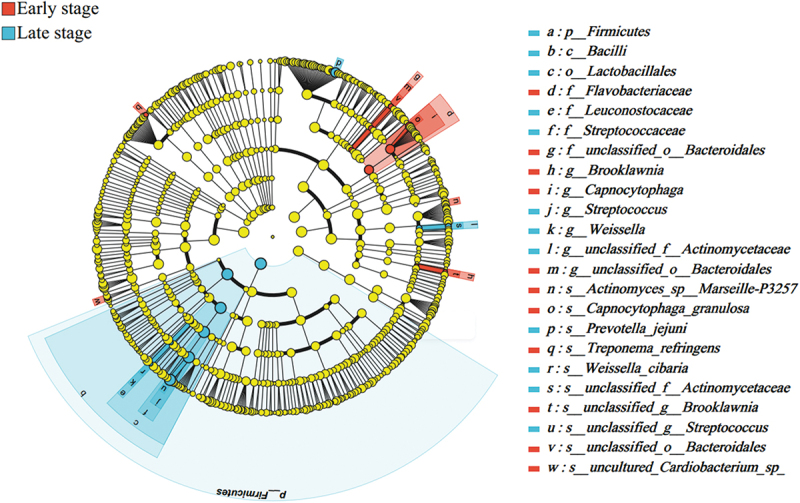
Lefse multilevel species difference discriminant analysis diagrams (from phylum to species) for T2DM duration. The color indicates a greater relative abundance of the microbial community in the group.

There was no significant difference between microorganisms in the HbA1c group. In the T2DM duration group, the genera *Streptococcus* and *Weissella* and the species *s__unclassified__g__Streptococcus*, *Prevotella_jejuni*, and *Weissella_cibaria* were significantly enriched in late stage group, and the genus *Capnocytophaga* and the species *Treponema_refringens and Capnocytophaga_granulosa* were significantly enriched in early stage group(Figure 3).

#### Correlation analysis

At the genus level, T2DM duration was positively correlated with *Streptococcus* (*r* = 0.369, *p* = 0.007) and negatively correlated with *Cardiobacterium* (*r* = −0.337, *p* = 0.014). The HbA1c level was not significantly correlated with the microbiome. At the species level, T2DM duration was positively correlated with *s_unclassified_g__Streptococcus* (*r* = 0.356, *p* = 0.010). HbA1c was positively correlated with *Streptococcus anginosus* (*r* = 0.304, *p* = 0.029).

#### Network analysis

**Figure 4.**
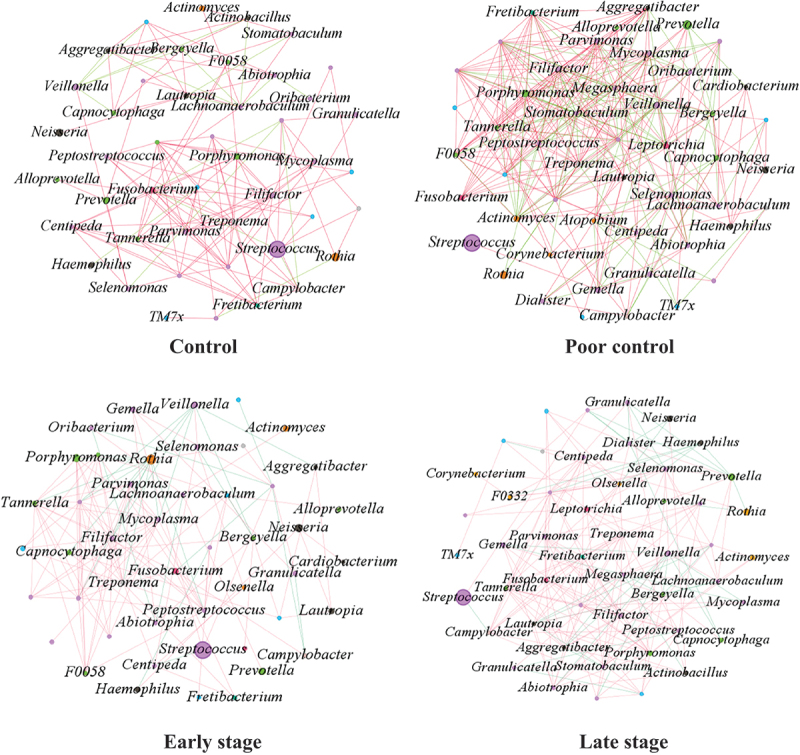
Microbial networks at the genus level for HbA1c (control and poor control) and T2DM duration (early stage and late stage). The size of the dots represents the abundance of the species, and the connecting line represents the correlation of the species, with a positive correlation in red and a negative correlation in green.

This study assessed the impact of HbA1c and T2DM duration on the interrelationships between the oral microbiome. The results revealed more complex interrelationships in the poor control group than in the control group (Figure 4) and more complex interrelationships in the late stage group than in the early stage group (Figure 4). In addition, we found that *Streptococcus* was significantly more abundant than the other genera, but its correlation with other microorganisms was very low.

## Discussions

Saliva samples were selected for sequencing in this study to explore the oral microbiome profile associated with different disease exposure and HbA1c control in elderly patients with T2DM. The oral cavity is a heterogeneous environment with a specific microbiome [35]. The microbiome of saliva is representative because of its ability to contact all parts of the mouth [36]. The collection of saliva samples is easy and noninvasive [35].

The elderly patients with T2DM in this study exhibited the highest abundance of *Firmicutes* and the fourth highest abundance of *Proteobacteria*. In contrast, younger patients with T2DM in China displayed a significantly greater abundance of *Proteobacteria* compared to *Firmicutes* [2]. This may be related to the fact that aging leads to a dysfunctional adaptive immune response that affects the salivary microbiome [5,27]. Omori et al. [5] reported a significant increase in the abundance of *Firmicutes* in a diabetic group compared with a healthy group. Most species of *Firmicutes* can produce endospores that resist dehydration and extreme environments, and many well-known pathogens can produce endospores [2]. A comparison with available microbiological data on T2DM in elderly individuals showed that at the phylum level, the top five phyla identified in the present study are in agreement with the findings of Omori et al. [5] At the genus level, the top five genera identified in the present study differed from those identified by Omori et al. [5] and Shaalan et al. [21] This may be due to racial differences, but the top genera identified in these studies were all *Streptococcus*. Some studies [12,37,38] have also shown that *Streptococcus* is a major source of oral microorganisms in T2DM patients. Saliva from T2DM patients in a high-sugar environment favours the growth of caries-associated acid-causing and acidophilic microorganisms [39], and many bacteria of the genus *Streptococcus*, such as *Streptococcus mutans*, *Streptococcus pyogenes* and *Streptococcus salivarius*, are closely associated with dental caries [3,40].

The initial manifestation of T2DM is insulin resistance, which progresses gradually to impaired glucose tolerance, hyperglycemia and complications [23]. Previous studies have shown that blood glucose levels can affect an individual’s oral microbiome. There was a clear reduction in the number of species in the impaired glucose tolerance and diabetes groups compared to the normoglycaemic group [41]. FBG levels were classified into three groups: normal (<6.1 mmol/L), high (6.1 ~ 7 mmol/L), and very high (>7 mmol/L), and the very high group showed both deterioration of the metabolic phenotype of the oral microbiome and ecological dysbiosis, with significant enrichment of *Leptotrichia, Staphylococcus, Catonella* and *Bulleidia* [42]. SG decreased salivary pH and decreased the ratio of *Firmicutes/Bacteroidetes* [22]. However, the above studies involved nondiabetic populations and only indicators of short-term diabetes status. Therefore, this study further investigated the influence of long-term indicators such as HbA1c, T2DM duration and T2DM complications on the oral microbiome in elderly T2DM patients, which was found to have little effect on the diversity and composition of the oral microbiome. There are greater levels of inflammatory and immune factor expression around dental implants in patients with fluctuating blood glucose than in patients with sustained hyperglycemia [43], suggesting that short-term fluctuations in blood glucose may have a greater impact on the oral microbiome.

The correlation between HbA1c levels and the oral microbiome is controversial. One study revealed that the abundance of *Prevotella nanceiensis* was negatively correlated with HbA1c levels [12]. Another study did not observe microbiomes that were significantly correlated with HbA1c levels, which is consistent with the results of this study [2]. The changes in T2DM are closely related to T2DM duration, disease staging and glucose control [44], whereas the correlation between T2DM duration and the oral microbiome has not been explored. At the genus level, correlation analyses revealed that the duration of T2DM was positively associated with *Streptococcus*, the most abundant species in the oral microbiome, and differential analysis revealed that *Streptococcus* was significantly more abundant in the late stage group than in the early stage group. *Streptococcus* is closely associated with a variety of inflammatory conditions and may be involved in the pathogenesis of T2DM [45]. Tiderencel et al. [46,47] reported that *Streptococcus* was positively associated with the risk of developing T2DM and may have an antiprotective effect against T2DM, indicating that increased *Streptococcus* abundance may be a marker of diabetes progression. At the species level, T2DM duration was positively associated with *the abundance of S_unclassified__g__Streptococcus*, and differential analysis revealed that *the abundance of S_unclassified__g__Streptococcus* was significantly greater in the late stage group than in the early stage group. Omori et al. [5] noted that some bacterial genera may be rarely detected in poorly controlled elderly T2DM patients but are unique; therefore, further large-scale studies limited to elderly individuals are needed to confirm this hypothesis.

Increased network edges and network nodes between the oral microbiome indicate a more stable oral microecology [47]. Previous studies have shown that oral microbiome interrelationships are simpler in T2DM patients than in healthy individuals [47]. In contrast, this study revealed that oral microbiome interrelationships became more complex as diabetes progressed. The above results suggest the possibility of a new relationship in oral microecology as T2DM progresses.

This study has some interesting findings on the impact of HbA1c control and T2DM exposure on the oral microbiome profile in the elderly population. However, this study has several limitations. First, this study has a cross-sectional research design with limitations in the interpretation of the results. Second, indicators of T2DM diagnosis, disease duration and complications were self-reported by patients in this study; however, older adults may be biased in reporting T2DM diagnosis due to memory loss and lack of professional knowledge. Third, this study measured the V3-V4 region of the 16SrRNA gene, which has limited precision in reflecting the species level of the microbiome. Finally, there is an effect of smoking on the oral microbiome; however, the results may have been influenced by the fact that smokers and nonsmokers were not included in the analysis in this study due to the large gap between their sample sizes, but overall, the number of smokers was small.

## Conclusions

*Streptococcus* is potentially one of the biomarkers associated with diabetes in elderly individuals due to its high abundance in diabetic patients, which is influenced by the course of diabetes and low interactions with other microorganisms. However, the present study has some limitations, and further studies are needed to break through these limitations and validate the results in the future.

## Supplementary Material

Supplemental Material

## Data Availability

The original contributions presented in the study are included in the article, further inquiries can be directed to the corresponding authors.
